# Light and the outcome of the critically ill: an observational cohort study

**DOI:** 10.1186/cc11437

**Published:** 2012-07-24

**Authors:** Ricardo A Castro, Derek C Angus, Seo Yeon Hong, Chingwen Lee, Lisa A Weissfeld, Gilles Clermont, Matthew R Rosengart

**Affiliations:** 1 The CRISMA Center (Clinical Research, Investigation, and Systems Modeling of Acute Illness), Department of Critical Care Medicine, University of Pittsburgh, 3550 Terrace Street, Pittsburgh, PA 15261, USA; 2 Escuela de Medicina, Departamento de Medicina Intensiva, Pontificia Universidad Catolica de Chile, Marcoleta 367, Santiago Centro, RM, 8330024, Chile; 3 Department of Surgery, University of Pittsburgh, 200 Lothrop Street, Pittsburgh, PA 15213, USA

## Abstract

**Introduction:**

Light before and during acute illness has been associated with both benefit and harm in animal models and small human studies. Our objective was to determine the associations of light duration (photoperiod) and intensity (insolation) before and during critical illness with hospital mortality in ICU patients. Based on the 'winter immunoenhancement' theory, we tested the hypothesis that a shorter photoperiod before critical illness is associated with improved survival.

**Methods:**

We analyzed data from 11,439 patients admitted to 8 ICUs at the University of Pittsburgh Medical Center between June 30, 1999 and July 31, 2004. Daily photoperiod and insolation prior to and after ICU admission were estimated for each patient by using data provided by the United States Naval Observatory and National Aeronautics and Space Administration and direct measurement of light gradient from outside to bedside for each ICU room. Our primary outcome was hospital mortality. The association between light and risk of death was analyzed using multivariate analyses, adjusting for potential confounders, including severity of illness, case mix, and ICU type.

**Results:**

The cohort had an average APACHE III of 52.9 and a hospital mortality of 10.7%. In total, 128 ICU beds were analyzed; 108 (84%) had windows. Pre-illness photoperiod ranged from 259 to 421 hours in the prior month. A shorter photoperiod was associated with a reduced risk of death: for each 1-hour decrease, the adjusted OR was 0.997 (0.994 to 0.999, p = 0.03). In the ICU, there was near complete (99.6%) degradation of natural light from outside to the ICU bed. Thus, light exposure once in the ICU approached zero; the 24-hour insolation was 0.005 ± 0.003 kWh/m^2 ^with little diurnal variation. There was no association between ICU photoperiod or insolation and mortality.

**Conclusions:**

Consistent with the winter immunoenhancement theory, a shorter photoperiod in the month before critical illness is associated with a reduced risk of death. Once in the ICU, patients are exposed to near negligible natural light despite the presence of windows. Further studies are warranted to determine the underlying mechanisms and whether manipulating light exposure, before or during ICU admission, can enhance survival.

## Introduction

Sunlight has profound effects on life. Although we are all generally aware of the sense of well-being induced by more sunlight, both more and less light trigger important adaptive changes in our underlying physiology. Seasonal physiologic adaptations in biological stress responses have been observed consistently in many animal species [1-4]. Superimposed are diurnal changes that are influenced by the duration of daily light exposure or photoperiod [5]. The consequences of these adaptive changes on critical illness are unclear. On the one hand, some studies have reported reduced mortality in patients with myocardial infarction and less delirium and post-operative pain in critically ill surgical patients after exposure to bright natural light or windows [6-8]. On the other hand, a shorter duration of light exposure prior to stress may promote survival. A non-visual optic pathway, responding to a shorter photoperiod, heightens the synthesis and release of melatonin and thereby enhances immunity [2,9,10]. Animals exposed to this short photoperiod exhibit marked resistance to endotoxemia, characterized by attenuated inflammation, earlier termination of the sick response, and improved survival: this is known as the winter immunoenhancement theory [11].

One limitation of prior clinical studies has been the absence of data quantifying and qualifying the exposure to light. Light is complex and is defined by photoperiod (that is, duration), insolation (that is, intensity), and wavelength. Few studies have incorporated this complexity into a study design. Contemporary medical monitors, continuous in use and ubiquitous throughout the intensive care unit (ICU), are rich in blue spectrum, which functions biologically as daylight [12-16]. Thus, the null results of more recent studies may stem from the near-constant, low-intensity, high-in-blue-spectrum lighting prevalent in ICUs. In this study, we wished to characterize the insolation and photoperiod of light exposure both prior to and during critical illness and determine the association of each with patient outcome. Our primary objective was to explore whether the duration or intensity of light, either prior to or during illness, is associated with outcome. In accordance with prior biological studies, we hypothesized that a shorter photoperiod is associated with improved survival.

## Materials and methods

### Study design

We conducted an observational retrospective cohort study, using the University of Pittsburgh Medical Center (UPMC) Acute Physiology and Chronic Health Evaluation (APACHE) III dataset. This dataset ran for many years and calculated APACHE III scores for every third patient. We linked data from this dataset to electronic demographic and clinical data from the HiDenIC (High-Density Intensive Care) database, which retrospectively collects information on patients admitted to the ICU at the UPMC main campus hospitals [17]. This study was approved by the Institutional Review Board of the University of Pittsburgh. Because all data were deidentified, consent was not required.

### Patients

Demographic and physiologic variables were available for 11,439 patients admitted to eight ICUs from 30 June 1999 to 31 July 2004. Of these patients, 9,534 had complete covariate data and were analyzed. To avoid the counting of multiple ICU outcomes for a single patient, only the first admission to an ICU for each patient was considered. The primary outcome variable was hospital mortality during the index admission. Many patients undergo elective scheduled ICU admission (for example, post-operative observation or extubation). Thus, we focused our analysis upon patients requiring more than 24 hours of ICU care.

### Sunlight exposure

We obtained photoperiod and insolation data from the US Naval Observatory (USNO) and the National Aeronautics and Space Administration (NASA). Precise photoperiod duration in hours and minutes and insolation, a measure of the solar energy striking a unit surface area in a unit time (kilowatt hours per square meter per day), were obtained at the exact longitude and latitude of UPMC Presbyterian University Hospital and Montefiore University Hospital (+40.447°N -79.9517°W) for each subject for each calendar day from 60 days prior to the period of hospitalization until the period ended. Variations of light in the hospital referral region and in the patient's primary residence were minimal. Thus, for pre-illness values, patients were assigned the photoperiod and insolation values of these coordinates.

For prior light exposure, we used photoperiod. Owing to a lack of information about a person's geographic location and real outdoor/indoor exposure, cumulative insolation could not be estimated accurately at the subject level. Both variables exhibited moderate collinearity: variance inflation factor (VIF) of 7, tolerance of 0.14, and conditional index of 32. Owing to the combined inability to accurately determine pre-illness insolation and the substantial collinearity, we restricted our analysis of pre-illness light exposure to photoperiod.

To determine light exposure once admitted to the ICU, we multiplied the raw NASA outdoor values for the main campus hospitals by room-specific light signal degradation ratios. These were adjusted at the ICU level to account for the signal degradation that occurs between the outside and the ICU bed, and the variation in sunlight exposure due to the geometric position of the ICU window relative to the sun. Outside and on-site light unit-specific measurements were performed with a digital photometer (RAC) with the window blinds open and then closed. Thus, an ICU-specific adjusted insolation value for each day of ICU hospitalization was calculated. An insolation value of zero was assigned to ICUs without windows.

### Variables and risk adjustment

Cumulative photoperiod prior to ICU hospitalization was defined as the total hours of daylight for the days preceding hospitalization. Our primary analysis was for cumulative 28-day photoperiod prior to ICU admission as evidence supports the view that measurable induction of immunoenhancement (for example, lymphocyte spontaneous blastogenesis) is first observed significantly after a 4-week exposure to altered photoperiod duration [18]. Secondary analyses were performed for 7- and 60-day cumulative photoperiods. Cumulative insolation after ICU admission was defined as total kilowatt hours per square meter per day after admission. Our primary analysis was for 24-hour insolation; we performed secondary analyses for 48- and 72-hour insolation. In each of these analyses, we excluded patients dying prior to the full period (for example, 24, 48, or 72 hours) of insolation.

The association between photoperiod and outcome was examined with photoperiod as a continuous variable, and the reference category was the highest cumulative 28-day photoperiod. We also categorized photoperiod into quartiles, and the reference category was the highest photoperiod quartile.

We addressed potential confounding due to variation in the case mix by controlling for the severity of illness and additional variables related to the outcome. The severity of illness was determined according to the APACHE III score (range of 0 to 299), and higher values indicated a greater severity of illness and risk of death on the day of ICU admission. Other risk-adjustment variables were age, sex, race (African-American, Caucasian, and other), the season of admission (defined by the equinoxes and solstices for each year as detailed by the USNO), the primary diagnosis at ICU admission (cardiovascular, pulmonary, gastrointestinal, trauma, sepsis, malignancy, renal failure, neuropsychiatric, immunodeficiency, and other), the location of the patient prior to ICU admission (home versus hospital), and the type of ICU (medical, surgical, coronary, cardiothoracic, or transplant).

### Statistical analyses

Univariate analyses of continuous and categorical variables were performed with Student *t *test and Pearson's chi-squared test as appropriate. We performed a random effects multivariate logistic regression to assess the association between cumulative photoperiod or adjusted ICU insolation and the risk of mortality, calculated crude and adjusted odds ratios (ORs) with 95% confidence intervals (CIs), and accounted for the correlation within ICUs and covariates specified *a priori *as potential confounders. For our primary analysis, the Pearson's test of goodness of fit was a *P *value of greater than 0.05. For each analysis, we included age, sex, race, season, admission diagnosis, ICU location, and APACHE III score. We ascertained the absence of serious collinearity between season and cumulative photoperiod by calculating VIFs and tolerance values, demonstrating a VIF of less than 5 and tolerance of greater than 0.2 in all circumstances. A separate analysis without adjustment for severity of illness was performed as acute changes in physiology (that is, APACHE III score) may or may not occur in the causal mechanism. We explored whether the association between light and mortality varied with season, race, or admission diagnosis by including an interaction term between light and these covariates. For the assessment of insolation and photoperiod as a continuous variable, we also used the fractional polynomial method, an iterative estimation process that determines the best-fitting polynomial-regression function [19]. This method makes no underlying assumptions about the relationship between photoperiod or insolation and outcome and thereby averts the potential bias involved in pre-specifying the functional form. By separating the period before ICU admission from the period during ICU admission, we also accounted for potential immortal time bias, a situation that refers to cohort follow-up time in a time-to-event analysis like our study; owing to the exposure definition, the outcome under study could not occur during the follow-up time [20]. Statistical analyses were performed with Stata 11.2 (StataCorp LP, College Station, TX, USA). A *P *value of less than 0.05 was considered significant.

## Results

In total, 11,439 index ICU admissions during the period of study were available for analysis. The final adjusted analysis included 9,534 patients admitted to eight ICUs at two hospitals. The cohort had an average APACHE III score of 52.9 and a hospital mortality of 10.7%. The characteristics of patients and seasons are shown in Table 1.

**Table 1:** Characteristics of patients according to season

Characteristic	Season				*P *value
	Spring (n = 2,566)	Summer (n = 2,367)	Fall (n = 2,275)	Winter (n = 2,326)	
Age, years^a^	60.1 ± 17.8	59.6 ± 18.0	60.4 ± 18.0	60.1 ± 17.6	0.48
Sex, percentage					
Male	58.9	58.6	59.7	58.7	0.89
Race, percentage					
Caucasian	79.1	81.5	81.0	79.1	0.04
African-American	14.0	11.3	11.9	14.2	
Other	6.9	7.2	7.1	6.7	
APACHE III score^a^	51.4 ± 25.2	51.5 ± 25.4	52.9 ± 26.0	52.6 ± 26.4	0.09
Diagnostic category, percentage					
Cardiovascular	28.1	28.2	31.9	28.8	0.01
Trauma	15.4	17.3	14.7	13.9	0.01
Gastrointestinal	9.5	8.3	9.5	9.2	0.41
Sepsis	6.5	5.4	7.1	6.8	0.09
Solid tumors	5.9	4.9	4.2	5.0	0.06
Neuropsychiatric	4.8	3.6	4.0	4.5	0.18
Pulmonary	4.1	3.7	4.6	4.2	0.50
Renal	1.0	1.1	0.9	1.1	0.87
Immunodeficiency	0.3	0.6	0.2	0.4	0.12
Other	24.6	26.8	23.0	26.1	0.01
Type of ICU, percentage					
Medical	32.9	33.1	29.8	32.9	0.05
Coronary care	10.7	9.6	11.7	9.9	0.07
Cardiothoracic	30.0	32.2	34.2	31.3	0.02
Surgical	6.2	6.3	7.2	5.4	0.07
Multidisciplinary	20.2	18.8	17.1	20.5	0.02
Length of stay, days^a^	4.8 ± 6.6	5.2 ± 9.2	4.9 ± 6.6	5.1 ± 6.9	0.97
Mortality, percentage	10.2	10.0	11.4	11.2	0.29

^a^Values are expressed as mean ± standard deviation. APACHE III, Acute Physiology and Chronic Health Evaluation III; ICU, intensive care unit.

### Pre-admission

**Figure 1:**
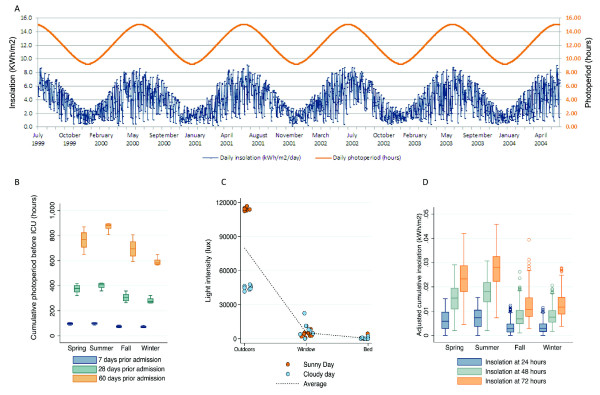
**Characteristics of insolation and photoperiod**. **(a) **Daily insolation and photoperiod from 30 June 1999 to 31 July 2004 (+40.447°N -79.9517°W). **(b) **Cumulative photoperiod before intensive care unit (ICU) admission, according to season. **(c) **Degradation of light signal (*lux*) from outdoors to ICU bed on sunny and cloudy days. **(d) **Adjusted cumulative ICU insolation (expressed in kilowatt hours per square meter) according to season.

**Figure 2:**
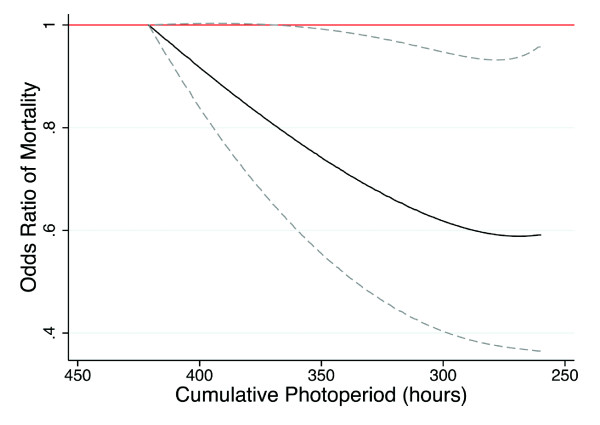
**Association between cumulative 28-day photoperiod and mortality**. The association between cumulative 28-day pre-admission photoperiod and in-hospital mortality was examined by using multivariate logistic regression and the fractional polynomial method. The adjusted odds ratio of death with decreasing cumulative 28-day pre-admission photoperiod is depicted. The reference category is the highest cumulative 28-day pre-admission photoperiod.

Seasonal variation in regional photoperiod and insolation are displayed in Figure 1a-d. The magnitude of and variation in cumulative pre-admission photoperiod were lowest during winter (Figure 1b and Table 2). After adjustment for severity of illness, age, sex, race, the season, diagnosis at admission, and ICU location, a 1-hour decrease in cumulative 28-day photoperiod was associated with a significant decrease in hospital mortality (adjusted OR 0.997, 95% CI 0.994 to 0.999; *P *= 0.03) (Table 3). Thus, in comparison with patients in the highest quartile of cumulative 28-day photoperiod, patients in the lowest quartile had a decreased risk of death (adjusted OR 0.69, 95% CI 0.47 to 1.01; *P *= 0.06). Figure 2 shows the effect of cumulative 28-day photoperiod when examined as a continuous variable in a multivariate model. Decreased mortality is seen throughout the distribution as photoperiod decreases from the highest to the lowest value. This relationship did not vary significantly by season, race, or admission diagnosis (interaction term between each covariate and photoperiod, *P *>0.10). Restricting our analysis to those patients admitted to the ICU within 24 hours of hospitalization did not alter these results (adjusted OR 0.996, 95% CI 0.993 to 1.000; *P *= 0.04). We also analyzed the effect of photoperiod without adjusting for APACHE III score since, according to the winter immunoenhancement theory, acute changes in physiology could be part of the causal mechanism (adjusted OR 0.998, 95% CI 0.0.995 to 1.001; *P *= 0.14) (Table 3).

**Table 2:** Cumulative photoperiod prior to intensive care unit admission according to season

Photoperiod	Season				*P *value
	Spring	Summer	Fall	Winter	
At 7 days	96.4 ± 6.8	98.3 ± 5.9	73.8 ± 6.6	72.2 ± 5.9	<0.001
At 28 days	374.8 ± 29.0	400.5 ± 19.4	306.1 ± 28.4	281.1 ± 19.2	<0.001
At 60 days	764.3 ± 65.7	870.9 ± 25.2	694.7 ± 63.9	589.8 ± 24.0	<0.001

Values are expressed as mean ± standard error of the mean. Photoperiod is expressed in hours.

**Table 3:** Association between cumulative 28-day photoperiod and outcome

Exposure	Odds ratio^a^	95% CI	*P *value
Photoperiod_28d _			
ALL^b^	0.997	0.994-0.999	0.03
ALL (-APACHE III)^c^	0.998	0.995-1.001	0.14
Photoperiod_28d _quartiles^b^			
First quartile	0.69	0.47-1.01	0.06
Second quartile	0.79	0.57-1.10	0.16
Third quartile	0.88	0.70-1.11	0.11
Fourth quartile	Referent		

^a^For each 1-hour increase in cumulative 28-day photoperiod. ^b^Adjusted for Acute Physiology and Chronic Health Evaluation (APACHE) III, age, sex, race, season, admission diagnosis, and intensive care unit (ICU) location. ^c^Adjusted for age, sex, race, season, admission diagnosis, and ICU location. CI, confidence interval.

We analyzed pre-admission exposure periods both shorter and longer than the 28-day threshold we hypothesized for biological effects. Decreasing cumulative 60-day pre-admission photoperiod continued to be associated with reduced risk of death (adjusted OR 0.998, 95% CI 0.997 to 0.999; *P *= 0.01). However, a significant association was not observed when examining a cumulative 7-day photoperiod (adjusted OR 0.989, 95% CI 0.977 to 1.001; *P *= 0.07). There was no association between season and risk of death (Tables 1 and 4). However, after adjusting for pre-admission photoperiod, both fall and winter were associated with an increased risk of death in comparison with spring (Table 4).

**Table 4:** Association between season and outcome

Exposure	Odds ratio^a^	95% CI	*P *value
Seasons^a^			
Spring	Referent		
Summer	1.01	0.82-1.25	0.90
Fall	1.08	0.88-1.33	0.46
Winter	1.08	0.87-1.33	0.49
Seasons^b^			
Spring	Referent		
Summer	0.93	0.74-1.16	0.52
Fall	1.35	1.01-1.81	0.04
Winter	1.46	1.03-2.08	0.03

^a^Adjusted for Acute Physiology and Chronic Health Evaluation (APACHE) III, age, sex, race, admission diagnosis, and intensive care unit (ICU) location. ^b^Adjusted for APACHE III, age, sex, race, admission diagnosis, ICU location, and cumulative 28-day photoperiod. CI, confidence interval.

### Intensive care unit

We observed an enormous degradation (>99.6%) of natural radiant light from outdoors to the ICU bed (Figure 1c). Thus, the exposure to natural light, once in the ICU, approached zero; the 24-hour insolation was 0.005 ± 0.003 kWh/m^2 ^and there was little diurnal variation (Figure 1d and Table 5). There was no association between ICU photoperiod or insolation and hospital mortality (data not shown).

**Table 5:** Adjusted cumulative intensive care unit insolation according to season

Insolation	Season				*P *value
	Spring	Summer	Fall	Winter	
At 24 hours	0.006 ± 0.004	0.007 ± 0.004	0.003 ± 0.002	0.003 ± 0.002	<0.001
At 48 hours	0.015 ± 0.006	0.018 ± 0.005	0.008 ± 0.004	0.008 ± 0.003	<0.001
At 72 hours	0.023 ± 0.007	0.027 ± 0.007	0.012 ± 0.005	0.012 ± 0.005	<0.001

Values are expressed as mean ± standard error of the mean. Insolation is expressed in kilowatt hours per square meter.

## Discussion

Our results bring attention to the complexity of light and emphasize the importance of quantifying and qualifying its dimensions in the context of clinical studies. We observed that, prior to admission, a shorter photoperiod is associated with a reduced risk of death among critically ill patients. This association persists after controlling for relevant confounders, a sensitivity analysis in which a variety of modeling assumptions were tested, and analyses that separately assessed light duration and intensity. However, once in the ICU, light exposure is negligible and lacking in diurnal variation. These observations suggest that light is relevant to human biology in the context of critical illness. Yet further study is needed to determine the potential utility of light manipulation as a meaningful intervention in health and disease.

Prior studies of myocardial infarction and critically ill surgical patients noted reduced mortality, delirium, and post-operative pain in patients exposed to bright natural light or windows while hospitalized [6-8]. The same effect has been observed in patients hospitalized for depression [7]. Newer studies have failed to replicate these findings [21]. We observed a near-complete loss of natural light exposure in the ICU. Thus, we were unable to meaningfully assess the association between insolation and outcome. These data and those of other studies also demonstrate the limited diurnal variation of light in the ICU [22]. Blue light, a predominant wavelength of modern light-emitting diode monitors, functions as daylight and most powerfully suppresses melatonin and cortisol secretion [12-14]. Coupled to the altered patterns of illumination inherent in '24-hour around-the-clock' ICU care, these mechanisms of perturbed light exposure may underlie the null results of other studies of the association between light, windows, and the outcome of ICU patients [21].

The underlying mechanism by which the photoperiod prior to admission would alter the outcome of critical illness is unknown. It is well known that the immune response to antigen differs quantitatively and qualitatively depending upon the duration of exposure to light [9,23]. In animal models, manipulating the day length modifies immunity toward either enhancement (shorter photoperiod) or relative depression (longer photoperiod), even during the winter [3,24-26]. Thus, the observed reduction in mortality with shorter photoperiods may be the consequence of adaptive alterations in immunity.

The winter immunoenhancement theory proposes that, during seasonal changes in environmental stress (that is, winter), selection favors individuals exhibiting enhanced immune function as a result of seasonal energetic redistribution and adjustments in reproduction, growth, and other responses [1,2,27]. It is important to appreciate that immunoenhancement is not synonymous with pro-inflammation. To the contrary, anti-inflammatory and regulatory immune functions are also enhanced. Thus, animals use photoperiodic information to anticipate and prepare for the energetically challenging conditions of winter. Similar mechanisms may be induced during the 'physiologic winter' of life-threatening illness and the accompanying massive energetic expenditures of abnormal protein metabolism, fatty acid degradation, and circulating inflammatory mediators [28]. Indeed, our results suggest that the human response to critical illness is conditioned by the predominant photoperiod as we observed reduced mortality with shorter photoperiods and longer nights. Determining the underlying mechanisms will require prospective longitudinal studies of the association between these characteristics of light and parameters of the inflammatory and neuroendocrine response to illness.

Season is strongly associated with the epidemiology of disease. Apart from photoperiod and immune responses, variations in day length and season are associated with relevant changes in ambient temperatures, humidity, biotic growth, food quality and quantity, water availability, and animal and human behavior, as well [4,29,30]. To capture these intangible factors in our model, we incorporated the composite variable of seasons. Winter and fall proved to be the seasons with highest mortality. We speculate that mortality may have been even higher in the absence of immunoenhancement. Indeed, after adjustment for the adaptive effect of cumulative photoperiod (for example, cumulative 28-day pre-admission photoperiod), the adjusted risk of death in winter was much greater than that in spring and summer.

Our study is limited by the accuracy of patient records and the reduced size of the cohort available for analysis due to random calculation of the severity score. We were unable to characterize individual-specific variations in sleeping patterns, regions of residence, and patterns of activity (indoors versus outdoors). Thus, we assumed that patients possessing the same day of ICU admission were exposed to the same cumulative photoperiod and insolation prior to ICU admission. These characteristics would have little effect on photoperiod duration (that is, the length of day), our primary exposure of interest and the one associated with mortality. We do not have biological patient data. Hence, the observed associations may be due to altered provider care rather than an effect on a specific biological process within the patient.

## Conclusions

Lower exposure to sunlight prior to critical illness appears to be associated with improved outcome. These adaptive changes are sensitive to induction throughout the whole year. Although an individual may abruptly become critically ill at any time of the year, many other events such as cardiopulmonary bypass and major operation can be anticipated. In such circumstances, a window of opportunity to influence patient outcome might exist. Once in the ICU, the predominant lighting exposure is of low intensity and limited diurnal variation. Future studies of light exposure after hospitalization will require artificial means of manipulating light insolation and wavelength to achieve levels possessing biological effects. The precise timing and characteristics of such an intervention remain to be elucidated. Nonetheless, validating these associations and understanding the underlying mechanisms may guide immune-modulating therapy or even the development of new therapeutic interventions.

## Key messages

Lower exposure to sunlight prior to critical illness appears to be associated with improved outcome.

Once in the ICU, the predominant lighting exposure is of low intensity and limited diurnal variation.

Manipulating light exposure prior to admission and during the initial period after admission may enhance survival for patients who are expected to receive a major biological insult.

The precise timing and characteristics of such an intervention remain to be elucidated.

## Abbreviations

APACHE: Acute Physiology and Chronic Health Evaluation; CI: confidence interval; ICU: intensive care unit; NASA: National Aeronautics and Space Administration; OR: odds ratio; UPMC: University of Pittsburgh Medical Center; USNO: US Naval Observatory; VIF: variance inflation factor.

## Competing interests

The authors declare that they have no competing interests.

## Authors' contributions

RAC, DCA, and MRR were involved in study conception and design, the acquisition and interpretation of data and data analysis, and drafting and revising the manuscript. SYH, CL, LAW, and GC were involved in study design, the acquisition of data and data analysis, and revising the manuscript. All authors read and approved the final manuscript.
